# Crinoline Dangers

**Published:** 1858-07

**Authors:** 


### CRINOLINE DANGERS.

Whereas, the ladies will be admired the world over, however fantastically or ridiculously they may dress. And,

Whereas, they will dress to suit themselves, being the actual sovereigns of creation—man being the second fiddle. And

Whereas, the loss of one of them is a public and private calamity: Be it therefore

*Resolved* unanimously, that our wives and daughters be seriously and frequently cautioned to guard against a terrible death by fire; and that if the dress becomes ignited, the most certain method of saving life is to lie down on the floor and roll over and over; or better still, draw the carpet over the body, head, and ears, this will instantly extinguish the flames, and prevent horrible and ghastly scars for life, about the face.

It is natural, in an accident of this kind, for one woman to run to the rescue of another, with self-sacrificing devotion; and the chances are, that both the rescued and the rescuer will suffer terribly. Have a little presence of mind, and enjoin the person on fire to lie down, but whether lying down or standing up, envelop the sufferer in a woollen shawl, or coat, or over-coat, or blanket from the bed, or the carpet or rug—any thing woollen. When the fire is extinguished, remove the clothing as speedily as possible, and cover every burned place with dry flour, the most universally accessible, the most instantaneous pain-arrester, and the most specially curative agent that can be employed. A moisture comes from the surface of the injured parts, which, mixing with the flour, makes a paste or glue impervious to the atmosphere. It is the oxygen of the atmosphere which *keeps up the burning*, after the flame is extinguished; so any means which excludes air arrests the burning and destruction. Thus it is that when a part is burned, the pain is instantly removed by plunging it in cold water, where it may be kept until the flour can be procured.

An estimable young lady of this city had her dress recently fired while passing a stove, the door of which was open; she died in great agony. A few days later, the daughter of a Boston gentleman was standing near the chimney-piece, when her dress took fire, and she died within a few hours. A London paper states, that within six weeks from January 1st, no less than *nineteen* deaths were recorded from fired garments. The greatest danger is from wood fires, and candles or lamps placed on the floor.

It is greatly to be regretted, that our wives and daughters will stultify themselves by a dress so useless, so unbecoming, so inconvenient, so extravagant, and so dangerous. One woman dresses in a manner to conceal the fact that she is soon to become a mother: and straightway every young girl in Christendom adopts the same style! It is a libel on our intelligence and our independence. This truckling aping of the frivolities of foreign fashion is a disgrace to the nation.

